# The influence of in ovo injection with the prebiotic DiNovo® on the development of histomorphological parameters of the duodenum, body mass and productivity in large-scale poultry production conditions

**DOI:** 10.1186/s40104-017-0176-2

**Published:** 2017-05-19

**Authors:** Adrianna Sobolewska, Gabriela Elminowska-Wenda, Joanna Bogucka, Agata Dankowiakowska, Anna Kułakowska, Agata Szczerba, Katarzyna Stadnicka, Michał Szpinda, Marek Bednarczyk

**Affiliations:** 1 Department of Animal Biochemistry and Biotechnology, University of Science and Technology in Bydgoszcz, Mazowiecka 28 Street, 85-084 Bydgoszcz, Poland; 2 Department of Normal Anatomy, the Ludwik Rydygier Collegium Medicum in Bydgoszcz, the Nicolaus Copernicus University in Torun, 24 Karłowicza Street, Bydgoszcz, 85-092 Poland

**Keywords:** Chicken, Duodenum, Histomorphological parameters, In ovo, Prebiotic, Productivity

## Abstract

**Background:**

Among various feed additives currently used in poultry nutrition, an important role is played by bioactive substances, including prebiotics. The beneficial effect of these bioactive substances on the gastrointestinal tract and immune system give rise to improvements in broiler health and performance nutrition, thus increasing the productivity of these birds. An innovative method for introducing bioactive substances into chickens is the in ovo injection into eggs intended for hatching. The aim of the study was to evaluate the development of histomorphological parameters of the duodenum and productivity in chickens injected in ovo with the prebiotic DiNovo® (extract of *Laminaria* species of seaweed, BioAtlantis Ltd., Ireland) on d 12 of incubation, under large - scale, high density poultry production conditions.

**Results:**

There was no significant impact of the injection of DiNovo® prebiotic on the production parameters of broiler chickens (body weight, FCR, EBI and mortality) obtained on d 42 of rearing. No significant impact of the DiNovo® injection on the duodenum weight and length was observed, as well as on the CSA, diameter and muscular layer thickness of the duodenum. The in ovo injection of DiNovo® significantly increased the width of the duodenal villi (*P* < 0.05) and crypt depth (*P* < 0.01) of chickens on d 21 of rearing. Other histomorphological parameters of duodenal villi at d 42 of chickens rearing such as: the height, width, and cross section area of villi were significantly greater in chickens from the control group compared to those from the DiNovo® group (*P* < 0.05 and *P* < 0.01).

**Conclusions:**

In conclusion, this study demonstrates that injection of DiNovo® prebiotic into the air chamber of egg significantly influences the histomorphological parameters on d 21 of rearing without negatively affecting productivity in chickens at the end of rearing.

## Background

Both the worldwide and domestic production of poultry meat has been increasing dynamically, and in 2014 Poland became the leader of that production in the EU [1]. Commercial breeding programs, balanced nutrition and good health status of the birds result in high effectiveness of poultry production. The parameters demonstrating an economic effect of rearing broilers are: FCR (Feed Conversion Ratio) and EBI (European Broiler Index).

A major factor affecting the efficiency of animal husbandry is proper nutrition that provides properly balanced nutrients. Feeding can affect not only the growth and development of the birds, but also - to some extent - the functioning of the immune system, primarily through the use of appropriate feed additives, such as various bioactive substances. Among various feed additives currently used in poultry nutrition, an important role belongs to prebiotics. Prebiotics have been shown to exert beneficial effects on the gastrointestinal tract of broilers [2] and to enhance feed efficiency, thus improving the productivity of these birds [3]. The use of prebiotics and probiotics in the diet of broilers and laying hens was a response to the prohibition of the use in feed antibiotic growth stimulators by the EU (Regulations (EC) No. 1831/2003 and 1334/2003).

Prebiotics are components of feed derived from sugars, including raffinose family oligosaccharides (RFOs), galactooligosaccharides (GOS) and ß-glucans that selectively stimulate both the growth and activity of the desired intestinal microflora [4]. ß-glucans are naturally occurring polysaccharides that can be synthesized by many prokaryotic and eukaryotic organisms [5]. These compounds may be a constituent of cell walls in plants, fungi and various microorganisms [6]. The prebiotic used in this study, DiNovo® (BioAtlantis Ltd., Ireland), is an extract of *Laminaria* spp. containing specific quantities of laminarin and fucoidan. Laminarins have shown promising immunomodulatory activities. Fucoidan was proven to have also antiviral and antibacterial properties which result in improved health, a lower mortality and enhanced productivity of animals [5, 7–10]. Both bioactives stimulated proliferation of beneficial microflora and improved digestibility of nutrients in monogastrics compared with non-fucoidan diet [11, 12]. The activity of prebiotics in the gastrointestinal tract is somewhat related to pH adjustment, which results in a beneficial effect on the composition of the intestinal microflora. To date, the methods of prebiotic supplementation used have been limited to administration with feed or water. An innovative method for introducing bioactive substances into chickens is the in ovo injection into eggs intended for hatching. This technique is based on the introduction - on the appropriate day of embryonic development - of bioactive substances into the air chamber of the egg or directly into the developing embryo [13]. A thorough understanding of various stages of the embryonic development in birds allows the optimal time of injection to be defined [14, 15]. The use of in ovo techniques to introduce prebiotics and probiotics to chickens provides a means of modulating the immune system at early embryonic stages. Substances administered in ovo during the embryonic development of birds reach the intestines and affect the development of the gastrointestinal tract before hatching. Villaluenga et al. [16] demonstrated that the optimal time for the injection of a prebiotic is the 12^th^ day of embryonic development. In comparison with injections on d 1, 8 and 17, a significantly higher number of bifidobacteria was observed in the gut. Moreover, on d 12, the chorioallantoic membrane is already fully developed and vascularized, while the embryo is surrounded by the amniotic fluid that remains in contact with the embryonic gastrointestinal tract, which allows the transport of substances from the air chamber into the intestine [17].

The intestine is highly specialized in the hydrolysis and absorption of nutrients, and constitutes the paramount barrier between the host’s external and internal environment. The integration of digestive, absorptive and immune functions of the gastrointestinal tract, as well as the ability to regulate these functions are of key importance for animals, including the productivity of livestock [18]. In the final phase of digestion and absorption of nutrients, a substantive role is played by intestinal villi lined with epithelium, composed of various cells [19]. The intestinal epithelium covering villi is invaginated into the lamina propria forming tubular glands called intestinal crypts. The crypts are comprised of populations of continuously proliferating stem cells. These cells are responsible for the formation of various types of intestinal epithelial cells. Among the most abundant cells are enterocytes that migrate to the top of villi and incorporate into it towards the intestinal lumen. These cells are responsible for the transport of nutrients from the intestinal lumen into blood vessels [20]. After several hours of life, enterocytes are replaced with new cells; while over time, the depleted cells peel off into the lumen.

The histomorphological parameters of all sections of the small intestine, such as the height of intestinal villi, the crypt depth and the ratio between these two values, are some of the indicators of the health and functional status of the intestine in chickens. An increase in height of intestinal villi and the appropriate ratio between the height of villi and crypt depth are a measure of the intensity of recovery processes of intestinal epithelial cells [21, 22]. Both shorter villi and deeper crypts lead to an increase in the secretion of digestive enzymes and to a decrease in the absorption of nutrients, and may result in a lower productivity of animals [21]. Simultaneous shortening of the villi and deepening of crypts may reduce the productivity of the flock because shorter villi reduce the total surface area of the intestinal absorption which results in poorer absorption of nutrients, and deeper crypt contributing to an increased secretion of digestive enzymes [23]. In contrast to mammals, the small intestine in birds is relatively shorter and the passage of the content is faster, therefore the digestive processes are more intense. Moreover, the supply of feed to the currently bred broilers, from the first d after hatching to the end of rearing, is often conducted 24 h a day. Therefore, thick muscular layers (*muscularis mucosae* and the intestinal *muscularis externa*) induce contractions of villi with longitudinal and transverse folds of the mucosa, thus permitting the appropriate motor activity of the intestine. This is accompanied by an enhanced use of nutrients through a more effective mixing of the intestinal content and a better contact with digestive enzymes resulting in the faster absorption of nutrients.

In terms of digestion, as the first loop of the small intestine, the duodenum is a very important one. The pancreas is located within this loop. The posterior end of the duodenum becomes the jejunum that subsequently passes into the ileum, but there is no clearly marked boundary between the jejunum and ileum [24]. Unlike in mammals, the avian duodenum does not include Brunner’s glands since the submucosa is particularly hypoplastic.

The aim of the study was to evaluate the productivity and development of the histomorphological parameters of the duodenum on d 21 and 42 of rearing in chickens injected in ovo with the prebiotic DiNovo® on d 12 of incubation.

## Methods

The experiment was conducted on Ross 308 broiler breeder eggs incubated in large - scale, high density commercial hatchery conditions (Drobex - Agro Ltd., Solec Kujawski, Poland) in Petersime incubators. On d 12 of incubation, eggs were candled, and the infertile ones or those containing dead embryos were discarded. A total of 54,000 eggs, containing living embryos were randomly divided into 2 equal groups: a control group and an experimental group, injected with the prebiotic DiNovo® (DiNovo® Group). DiNovo® is an extract of *Laminaria* spp. containing laminarin and fucoidan (BioAtlantis Ltd., Ireland). The control group was injected with 0.2 mL of sterile physiological saline, while the eggs of the experimental group were injected with DiNovo®, 0.88 mg/egg dissolved in 0.2 mL of physiological saline. The solutions were delivered into the air chamber of every egg, and the hole in the egg shell was sealed with an automatic system dedicated for the in ovo injection of prebiotics.

### Animals

After hatching, the chickens were reared on the same farm in separate broiler houses (with the same environmental conditions) and fed on commercial diets (starter, grower, finisher) for 42 d. They were fed on and watered ad libitum. Either group (control group - CG, and experimental group - DiNovo® group) consisted of 25,000 chickens. The rearing experiment was conducted on the experimental farm of the Drobex - Agro company and lasted for 42 d, upon the approval of the Polish Local Ethical Commission (No 22/2012. 21.06.2012) and in accordance with the animal welfare recommendations of the European Union directive 86/609/EEC, providing adequate husbandry conditions with continuous monitoring of stocking density, litter, ventilation etc.

The cumulative feed intake for the whole period of rearing was measured and feed conversion ratio (kg feed intake/kg live mass gain) was calculated. The European Broiler Index (EBI) according to the following formulae was also calculated:

$$ \mathrm{E}\mathrm{B}\mathrm{I}=\frac{\mathrm{Viability}\ \left(\%\right) \times \mathrm{ADG}\ \left(\mathrm{g}/\mathrm{chick}/\mathrm{d}\right)}{\mathrm{FCR}\ \left(\mathrm{kg}\ \mathrm{feed}/\mathrm{kg}\ \mathrm{gain}\right) \times 10} $$

ADG = average daily gain

Viability (%) = chicks remaining at the end of period (%)

On the day of slaughter (d 42 of life), all chickens from both groups were transported to the Drobex - Agro slaughterhouse, and their mean body weight was calculated before slaughter in accordance with the methodology and technology used in that establishment.

### Histomorphological samples

The material for the morphological and histological analysis of the duodenum was collected from 21- and 42-day-old chickens of each. Before slaughter, a total of 100 chickens (a representative selection) from each group were weighed, and their mean body weight was calculated. Subsequently, 15 chickens per group, with the body weight similar to the mean for the group were selected. After slaughter, the small intestine was removed out and the duodenum was dissected, measured and weighed. Samples for histomorphometrical analyses (approx. 2 cm) were taken from the midway of the duodenum.

### Histomorphological examination

The sampled segment of the duodenum was carefully washed with 0.9% saline and then fixed in 4% formalin buffered with CaCO_3_. The fixed samples were dehydrated, cleared and permeated with paraffin in a tissue processor (Thermo Shandon, Chadwick Road, Astmoor, Runcorn, Cheshire, United Kingdom), and subsequently embedded in paraffin blocks using an embedding system (Medite, Burgdorf, Germany). Thus, formed blocks were cut on a rotary microtome (Thermo Shandon, Chadwick Road, Astmoor, Runcorn, Cheshire, United Kingdom) into slices of 10 μm thick which were successively placed on microscope slides coated with ovoalbumin with an addition of glycerol.

### Staining methods

Before staining, the specimens were deparaffinized and rehydrated. The specimens were then stained using the periodic acid - Shiff reagent (PAS) method for the morphometric analysis of the duodenum.

### Histomorphological measurements

Using a Carl Zeiss microscope (Jena, Germany) equipped with a ToupCam^TM^ digital camera and the MultiScan 14.02 computer software for microscopic image analysis (Computer Scanning Systems II, Warsaw, Poland), the following measurements were done: the height and width of intestinal villi and the depth of intestinal crypts. The measurement of the height of intestinal villi was conducted on 10 duodenal villi per one individual. The height was measured from the top to the base of the villus at the entrance to the intestinal crypt. The width of the villus was measured at half of its length. Subsequently, the villus surface area was calculated using the formula proposed by Sakamoto et al. [25]: (2π) × (VW / 2) × (VH), where VW = villus width, and VH = villus height. The crypt depth was defined as the depth of the invagination between adjacent villi. This parameter was measured in10 crypts [26].

In order to measure and calculate the thickness of the muscular layers and the cross - sectional area (CSA) of the duodenum, microscopic slides were imaged on a Kaiser rePro image capture system using a Canon EOS 70D digital SLR camera equipped with a Canon 100 mm f/2.8 L EF MACRO IS USM lens. For the calculation of the above listed parameters, the NIS ELEMENTS AR software (Nikon, Japan) was used. System precalibration was based on the microscopic reference line captured in the same conditions as the analyzed slides. Linear measurements of the thickness of the muscular layer of the duodenum were conducted on three consecutive slices of that segment by selecting two extreme points. The cross sectional area (CSA) of the duodenum was estimated on the base of the ellipse automatically generated from 5 different points localized on the circumference of its tunica muscularis. The diameter of the duodenum was calculated based on the cross - sectional area measured.

### Statistics

The obtained results were subjected to one - way analysis of variance (body weight before slaughter, mortality, FCR, EBI) and two-way analysis of variance (histomorphological measurements of the duodenum) using the SAS Institute Inc. 2013 computer program. SAS/STAT(r) 9.4 User\’s Guide. Cary, NC: SAS Institute Inc. The arithmetic mean ($\overline{x}$) and standard deviation (SD) were calculated. The significant differences between groups were tested using Duncan’s multiple range test.

## Results

Table 1 presents the evaluation results of the production parameters of broiler chickens (body weight before slaughter, FCR, EBI and mortality) obtained on d 42 of rearing. There were no significant differences in the case of the above mentioned parameters between the control and DiNovo® group. However, considering such a large number of broiler chickens in the experience, we can talk about the tendency a more favorable impact of the DiNovo® prebiotic injected at 12 d of incubation for the production traits of studied birds. The experimental group indicated a greater body weight before slaughter and the EBI ratio and a lower rate of the FCR ratio compared to the control group.

**Table 1:** Productivity parameters of chickens on d 42 of rearing

Parameters	D 42		*P*-value
	Control	DiNovo®	
Body weight before slaughter, g, *n* = ^a^	2140	2210	0.1998
Mortality, %	4.29	4.36	0.5970
FCR	1.79	1.72	0.3041
EBI	288	308	0.0971

^a^Control group, *n* = 21,934 individuals

DiNovo® group, *n* = 22,980 individuals

**Fig. 1:**
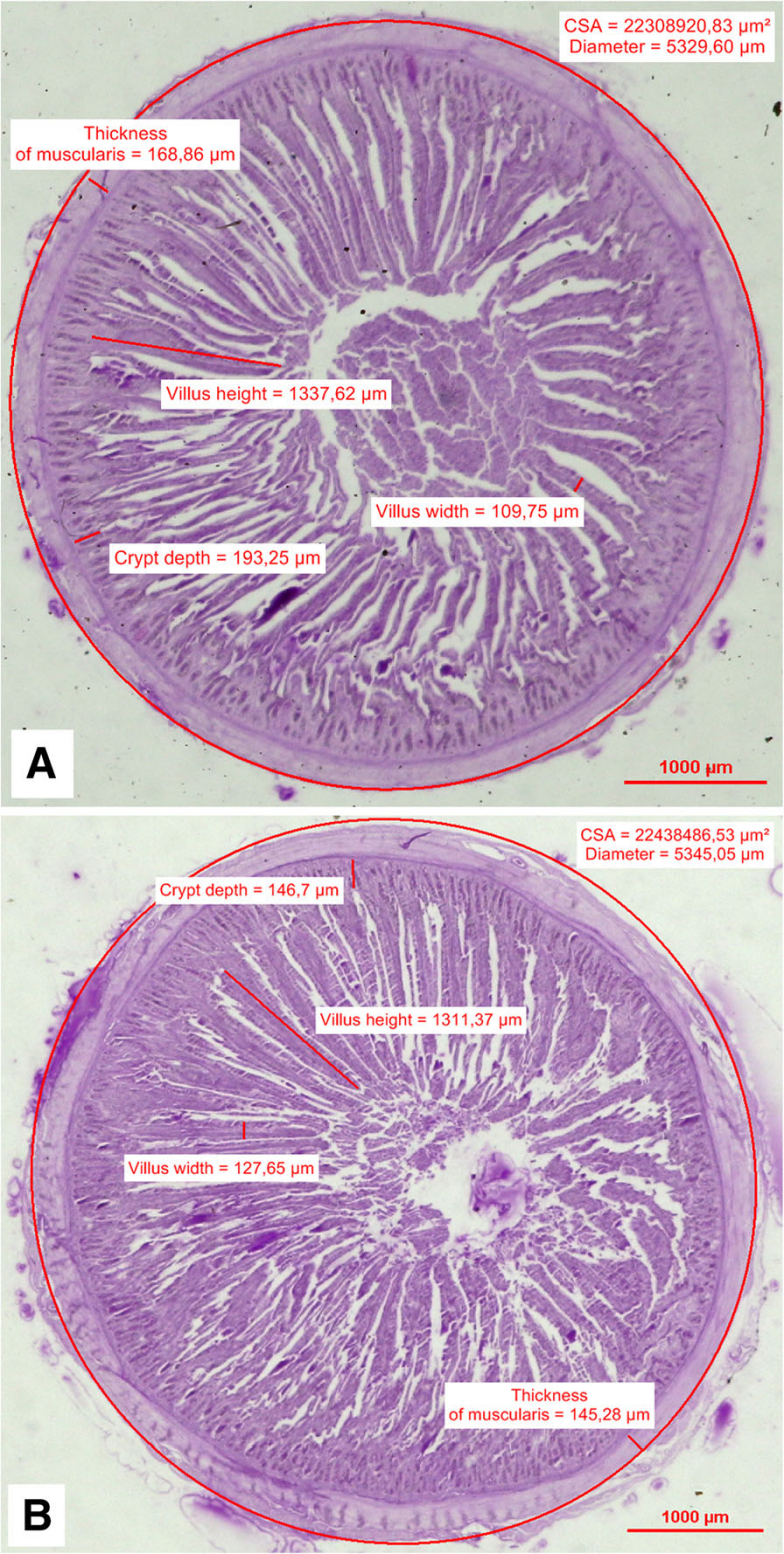
**a** Photomicrograph (light microscope) of the duodenum: CSA, diameter, thickness of muscularis and villus height, villus width, crypt depth in the control group on d 21. **b** Photomicrograph (light microscope) of the duodenum: CSA, diameter, thickness of muscularis and villus height, villus width, crypt depth in the DiNovo® group on d 21

**Fig. 2:**
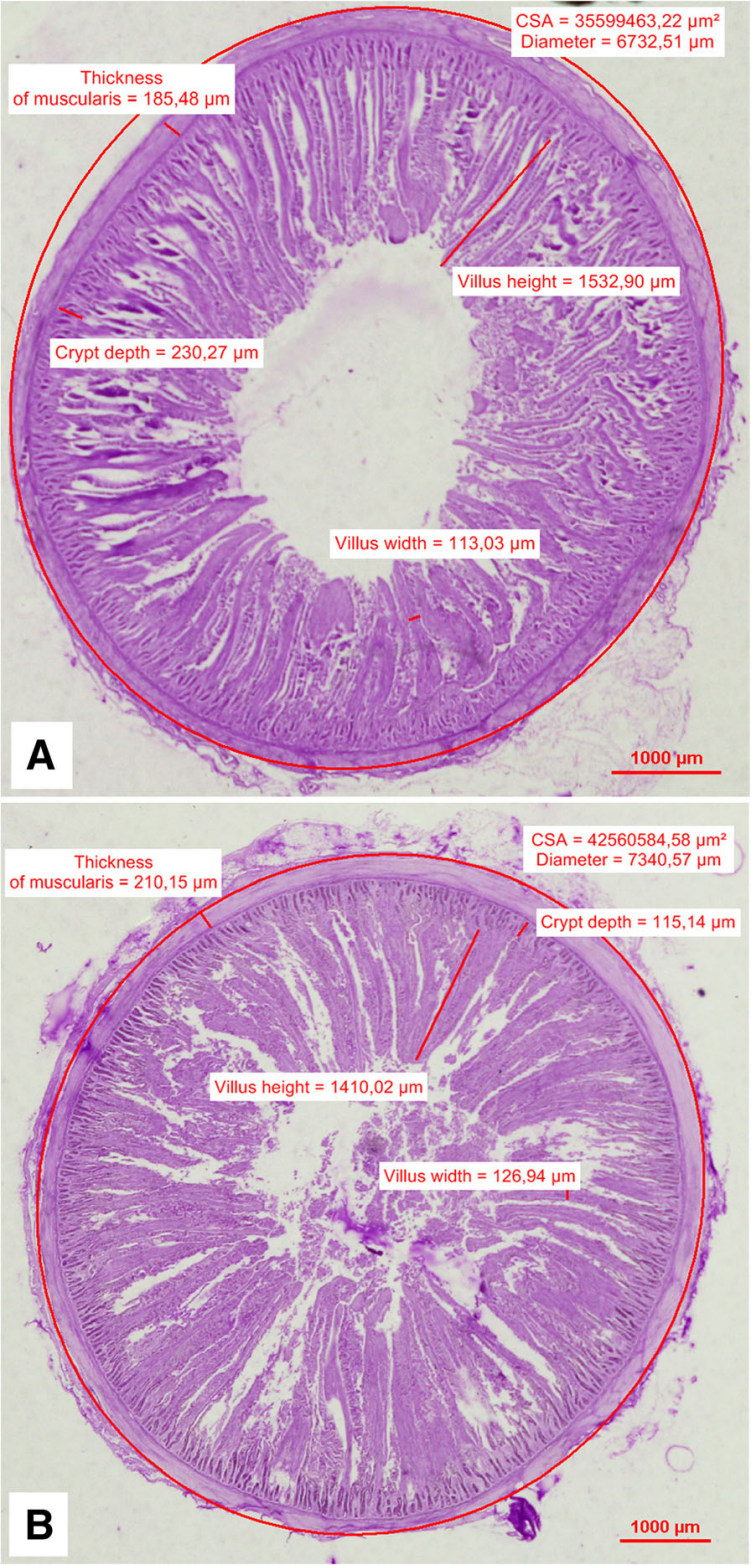
**a** Photomicrograph (light microscope) of the duodenum: CSA, diameter, thickness of muscularis and villus height, villus width, crypt depth in the control group on d 42. **b** Photomicrograph (light microscope) of the duodenum: CSA, diameter, thickness of muscularis and villus height, villus width, crypt depth in the DiNovo® group on d 42

Table 2 presents the mean body weight of chickens for histological studies and the morphological parameters of the duodenum on d 21 and 42 of rearing in chickens from both investigated groups. The mean body weight values in the control and DiNovo® groups were similar in both terms of slaughter and did not differ significantly. No significant impact of the DiNovo® injection on the duodenum weight and length was observed, as well as on the CSA, diameter and muscular layer thickness of the duodenum (Fig. 1a, b; Fig. 2a, b).

**Table 2:** Body weight of the chickens (g) and histomorphological measurements of the duodenum on d 21 and 42 of rearing

Parameters	D 21		D 42	
	Control	DiNovo®	Control	DiNovo®
Body weight of the chickens with histological samples taken, g, *n* = 15	811 ± 23.2	845 ± 15.1	2170 ± 30.3	2190 ± 51.6
Duodenal weight, g	7.40 ± 0.2	8.03 ± 0.4	15.30 ± 0.6	15.28 ± 0.6
Duodenal length, cm	25.79 ± 3.9	25.30 ± 3.0	33.60 ± 2.6	33.16 ± 3.2
Duodenal CSA (cross-sectional area), mm^2^	23.81 ± 4.27	23.73 ± 2.82	35.34 ± 4.66	36.62 ± 6.14
Duodenal diameter (excluding tunica serosa), μm	5484 ± 493.94	5484 ± 330.71	6690 ± 444.08	6808 ± 537.65
Muscularis thickness, μm	148 ± 25.38	149 ± 22.15	194 ± 13.50	191 ± 31.31
Villus height, μm	1316 ± 43.2	1302 ± 43.2	1536^a^ ± 56.8	1383^b^ ± 32.3
Villus width, μm	109^b^ ± 3.6	126^a^ ± 5.7	115^A^ ± 5.5	99^B^ ± 2.3
Villus surface area, μm^2^	443,997 ± 13.654	514,529 ± 34.306	558,730^A^ ± 41.777	429,764^B^ ± 11.648
Crypt depth, μm	108^B^ ± 4.2	146^A^ ± 1.8	227^A^ ± 3.7	113^B^ ± 4.6

Values with different letters differ significantly between treatments (a-b *P* < 0.05, A-B *P* < 0.01)

The histomorphological parameters of duodenal villi on d 21 and 42 of rearing are also presented in Table 2 and Figs. 1a, b and 2a, b. The in ovo injection of DiNovo® significantly increased the width of duodenal villi of chickens on d 21 of rearing (*P* < 0.05). This resulted in a greater surface area of villi in these birds, however this was not confirmed statistically. DiNovo® prebiotic, used in the study, significantly increased the crypt depth on d 21 (*P* < 0.01), in contrast to the last day of rearing, wherein crypts in the experimental group were significantly shorter in comparison to the control group (*P* < 0.01). Other histomorphological parameters of duodenal villi at 42 d of chickens rearing such as: the height, width, and surface area of villus were significantly greater in chickens from the control group compared to those from the DiNovo® group (*P* < 0.05 and *P* < 0.01).

Figure 1a, b; Fig. 2a, b present microscopic images of the cross sections of the duodenum on d 21 and 42 of rearing in the chickens from the control group and the DiNovo® group. The muscular layer thickness, CSA (ellipse), diameter of the duodenum, height and width of the intestinal villi, and crypt depth are marked in each image.

## Discussion

In the present study, the effect of in ovo injection of the prebiotic DiNovo® on the morphometric parameters of the duodenum in broiler chickens on d 21 and 42 of rearing was examined. The obtained results are problematic to compare with the literature data because most studies have been focused on the impact of prebiotics as additives in feedstuffs, and not administered in ovo, as achieved in this study [27–31]. Only Tako et al. [32] and Cheled-Shoval et al. [33] studied the effect of various substances injected during chicken embryogenesis on small intestine morphometry. The first group injected in ovo into the amniotic fluid carbohydrates and β-hydroxymethylbutyrate (HMB) on d 17.5 of egg incubation (19E – d of embryonic development, 20E, hatch and 3 d). The authors observed an increased surface area of the intestinal villi at all tested time points of the embryonic development and on d 1 and 3 after hatching in chickens provided with HMB. In turn, the smallest surface area of intestinal villi was observed in the control group with 1.5 to 3 times lower values than in the group receiving HMB. In our study we have not achieved such a positive effect on the last day of rearing chickens (42 d of rearing). There was, however, a clear tendency of beneficial effects of used prebiotic on the surface area of duodenal villi at d 21 of life. We can therefore assume that the effect of the DiNovo® injection occurs in an earlier period of rearing chickens that was also observed in the study by Bogucka et al. [34], who analyzed the effect of various bioactive substances given in ovo on the histomorphology of chickens small intestine in the first days after hatching. Similarly, Cheled-Shoval et al. [33] used the in ovo technique 3 d before hatching to administer a preparation of mannan oligosaccharide (MOS), and examined the morphology of the intestine of chickens (Cobb 500) on d 1 after hatching. These authors observed a positive impact of the prebiotic on the height and width of intestinal villi and the depth of intestinal crypts. However, in our study a significant effect (*P* < 0.05) of in ovo injection of DiNovo® on the width of duodenal villi and crypt depth (*P* < 0.01) on d 21 of rearing compared to the control group has been found. Significantly deeper crypts in this group may indicate intense renewal of the intestinal epithelial cells, which in turn can exert a positive effect on the function of the intestinal absorption and secretion. Evaluating the impact of the MOS on the intestinal muscular layer by Cheled-Shoval et al. [33], the authors found that the thickness of the muscular layer was significantly greater (*P* < 0.05) in chickens from the experimental group (MOS) compared with the control group. However, in our study there were no significant differences in the thickness of muscular layer in the duodenum in both on d 21 and 42 of chickens life (Table 2).

A study by Houshmand et al. [35] focused on the effect of the prebiotic MOS (Bio-MOS) and a probiotic (*Bacillus subtilis* and *Clostridium butyricum*) on the morphology of the duodenum and jejunum in cockerels (Cobb) on d 21 and 42 of rearing. The study revealed significantly higher villi in the duodenum in 21-day-old chickens that received the prebiotic with feed (Starter), as compared with the chickens from the control group and those receiving the probiotic. No statistically significant differences in the crypt depth were observed between the study groups. The same results were obtained in cockerels on d 42 of life. Similarly to our study, Houshmand et al. [35] did not report significant differences in either the length of the duodenum or the weight of the small intestine and duodenum (% of body weight) on both d 21 and d 42 of rearing. However, the duodenum on d 21 and 42 was approximately by1 cm and 4 cm shorter, respectively than in this study.

The analysis of the morphometric results in the current study (Table 2) with reference to the productivity parameters (Table 1), i.e. body weight on slaughter and FCR does not produce clear results. It seems that by d 21 of rearing, the wider villi and deeper crypts in the DiNovo® group positively affected the digestive and absorptive potential of these chickens. Perhaps, in the second half of rearing, despite the significantly poorer histomorphometrical properties of the duodenum in the DiNovo® group, the better digestive and absorptive potential from the first period of life of these chickens (up to d 21) contributed to the final productivity obtained in this group. As a result, the DiNovo® group indicated their body weight before slaughter greater by 70 g, FCR improved by approximately 0.07 units, and EBI greater by 20 points. Taking into account the fact that the values of these parameters were calculated in over twenty thousand chickens from each group, the obtained results are reliable and have a high implementation value. The apparent discrepancy between the productivity parameters and the morphology of the duodenum on d 42 may be due to the lack of data on the morphology of further sections of the intestine (jejunum and ileum). Furthermore, the morphometric parameters of the small intestine are characterized by a relatively high intra-species variability and high dynamics fettered by numerous factors.

## Conclusions

In conclusion, this study demonstrates that injection of DiNovo® prebiotic into the air chamber of egg significantly influences the histomorphological parameters on d 21 of rearing without negatively affecting productivity in chickens at the end of rearing.

## Abbreviations

ADG: Average daily gain
CSA: The cross - sectional area
E: Day of embryonic development
EBI: European Broiler Index
FCR: Feed Conversion Ratio
GOS: Galactooligosaccharides
HMB: β-hydroxymethylbutyrate
MOS: Mannan oligosaccharide
PAS: Periodic acid–Schiff
RFO: Raffinose family of oligosaccharides
SD: Standard deviation
